# Exploring Therapeutic Potential of *Pleurotus ostreatus* and *Agaricus bisporus* Mushrooms against Hyperlipidemia and Oxidative Stress Using Animal Model

**DOI:** 10.3390/foods13050709

**Published:** 2024-02-26

**Authors:** Touseef Iqbal, Muhammad Sohaib, Sanaullah Iqbal, Habib Rehman

**Affiliations:** 1 Department of Food Science and Human Nutrition, University of Veterinary and Animal Sciences, Lahore 54000, Punjab, Pakistan; dr.touseefiqbal@gmail.com (T.I.); sanaullah.iqbal@uvas.edu.pk (S.I.); 2 Department of Physiology, University of Veterinary and Animal Sciences, Lahore 54000, Punjab, Pakistan; habibrehman@uvas.edu.pk

**Keywords:** hypercholesterolemia, *Pleurotus ostreatus*, *Agaricus bisporus*, dyslipidemia, antioxidants, phytochemicals

## Abstract

The mushrooms oyster (*Pleurotus ostreatus*) and white button (*Agaricus bisporus*) contain bioactive compounds that have potential beneficial effects on hypercholesterolemia and cardiovascular diseases. In this study, hypolipidemic and antioxidative potential of these mushrooms’ extract were explored using hypercholesterolemic (HC) rats as animal model. For the study, 56 adult rats were divided into seven groups, i.e., G_1_ (negative control), G_2_ (positive control group), G_3_ (HC rats with statin drug orally), G_4_ and G_5_ (HC rats @ 100 and 200 mg/kg body weight (BW) dose of oyster mushroom extracts), and G_6_ and G_7_ (HC rats @ 100 and 200 mg/kg BW dose of white button mushroom extracts). The hypercholesterolemia was induced experimentally in fasted rats through a high-fat diet along with injection of triton WR-1339. After 48 h, the treatment groups were given extract for 28 days along with standard diet. At the trial termination, we analyzed the blood sugar levels, antioxidant parameters, lipid profile, and renal function, as well as conducting liver function tests of the rats. The results indicated that positive control group rats exhibited increased levels of total cholesterol (TC), triglycerides (TG), low-density level (LDL), and very-low-density level (vLDL) by 19%, 37%, 52%, and 32%, respectively, and 53% decrease in HDL, whereas treatment groups that received 200 mg oyster and white button mushroom extracts reported 15%, 34%, 22% reduction in TC, TG, vLDL, respectively, and 22% improvement in HDL level. The enzyme profiles of different groups showed non-significant differences, although both mushroom extracts provision reduced glutamic oxaloacetic transaminase (GOT) and glutamic pyruvic transaminase (GPT) levels. Overall, the results indicated that mushroom extracts were helpful in maintaining oxidative stress and have the potential to improve dyslipidemia in the tested rat animal model.

## 1. Introduction

A healthy and balanced diet having nutrient-rich foods provides the essential nutrients like vitamins, minerals, proteins, carbohydrates, and fats required for optimal human health. Hypercholesterolemia is one of the leading causes of morbidity and mortality in Western countries and the Asia–Pacific region [1]. Asia has been considered with higher mortality and morbidity due to non-communicable diseases like dyslipidemia, obesity, and diabetes burden, and Pakistan is not an exception in the scenario. Studies have revealed that elevated levels of cholesterol, triglycerides, and low-density lipoproteins, along with lower levels of high-density cholesterol, are the major risk factors of arthrosclerosis leading to ischemia and cardiac diseases in humans. Epidemiological research has suggested that hypercholesterolemia is a major risk factor for other non-communicable diseases like diabetes and CVD [2]. According to the World Health Organization (WHO), the prevalence of hyperlipidemia and hypercholesterolemia are increasing in the world and about 2.6 million deaths are caused by hypercholesterolemia disorders as it leads to several chronic diseases like diabetes and cardiovascular disorders [3,4]. In Asian countries like Pakistan, the prevalence of dyslipidemia is even higher owing to the poor lifestyle habits as well as lack of provision of a healthy diet. A study reported the prevalence of dyslipidemia in Pakistani population as 48.9% which is also alarming considering the burden of non-communicable diseases [5].

Hyperlipidemia, in reality, reflects elevated lipids (fats) level in blood, especially including cholesterol and its types, as well as triglycerides. Cholesterol, which is a waxy, fat-like substance present in cells considered essential for bodily functions such as hormones production, vitamin D, and substances that help to digest food. However, high levels of cholesterol in blood elevate the risk of cardiovascular diseases like heart stroke, etc. Hyperlipidemia can be categorized into two types: mainly, hypercholesterolemia that is characterized by high levels of cholesterol in the blood. The high levels of LDL cholesterol are associated with an increased risk of atherosclerosis (buildup of plaque in the arteries), while higher levels of HDL cholesterol are generally considered protective against cardiovascular diseases. However, hypertriglyceridemia refers to elevated levels of triglycerides in the blood and theses fats are often associated with the consumption of excess calories, particularly from carbohydrates and fats, and linked to an increased risk of cardiovascular diseases. Hyperlipidemia can be caused by a combination of genetic and lifestyle factors. Poor diet, lack of physical activity, obesity, smoking, excessive alcohol consumption, and certain medical conditions (such as diabetes and kidney disease) can contribute to the development of hyperlipidemia. In some cases, genetic factors can also play a significant role. The management of hyperlipidemia typically involves lifestyle modifications along with medication as per need; however, lifestyle modification changes include adopting heart-healthy diet rich in fruits, vegetables, whole grains, and lean proteins, and minimizing saturated and trans fats, adopting regular physical activity and weight management, and avoiding smoking and excessive alcohol consumption. Managing hyperlipidemia is important for reducing the risk of cardiovascular diseases and promoting overall heart health [6].

Edible mushrooms are considered as an important nutritious food having multiple health benefits as mushrooms are a good source of important phytochemicals, antioxidants, phenolic compounds (derivatives of benzoic acid or cinnamic acid), flavonoids, and terpenoids (monoterpenoids and diterpenoids). Phytosterols (ergosterols, sitosterol, campesterol, and stigmasterol) are plant sterols that have cholesterol-lowering properties [6]. Phytosterols (ergosterols, sitosterol, campesterol, and stigmasterol) are plant sterols that have cholesterol-lowering properties. The bioactive and phytochemical composition of mushrooms provide various health benefits, such as being antidiabetic, antioxidant, anti-inflammatory, and hypoglycemic, as well as producing hypocholesterolemia effects in the living organism [7].

White button mushrooms, scientifically known as *Agaricus bisporus*, are one of the most common, widely consumed mushrooms in the world and exhibit significant therapeutic potential with the presence of lipid polyphenols, steroids and steroid derivatives, lipid polyphenols, beta-glucans, and antioxidants. Ergostane steroids of button mushrooms exhibit significant potential in reduction in lipid profile [8]. Strong reducing power, scavenging activity, and lipid peroxidation inhibition, and the presence of phenolics (p-hydroxybenzoic acid, p-coumaric acid, and cinnamic acid) and plant sterols can suggest adding white button mushrooms among dietary approaches to managing hypercholesterolemia [9].

The oyster mushroom (*Pleurotus ostreatus*), the second most cultivated mushroom, followed by the white button mushroom, is recognized for its potential to manage dyslipidemia [10]. Being rich in polyphenols (sesquiterpenoids and diterpenoids) and fatty acyls (alcohols), and other bioactive compounds, oyster mushrooms are identified in the category of antioxidant and therapeutic diet for dyslipidemia [11]. The compositional concentrations of phytosterols, ergothioneine, and polyphenolic compounds in oyster mushrooms can play a beneficial role in controlling elevated cholesterol [12]. The strong antioxidant properties of oyster mushrooms can contribute to controlling oxidative stress. Furthermore, mushrooms can be incorporated into the diet as a natural and effective strategy for managing dyslipidemia and promoting cardiovascular health [13].

The rising consumption of edible mushrooms in Pakistan is owing to the country’s increased demand for a rich nutritional profile as well as the fact that there have been some processing plants established in the country for mushrooms but there are no comprehensive data that could potentially explain the benefits of consumption of these indigenous mushrooms for therapeutic potential, particularly with reference to managing hypercholesterolemia and diabetes. Considering the scenario, this experimental design study was conducted to investigate and compare the hypolipidemic and antioxidant potential of white button and oyster mushroom extracts using rat animal model to explore possible potential health benefits of these mushrooms in managing lipid disorders and oxidative stress-related conditions.

## 2. Materials and Methods

### 2.1. Plants and Chemical Materials

The analytical kits for cholesterol FS 10, glucose GOD FS 10, and alanine transaminase (ALT) (GPT) FS (IFCC mod.), creatinine FS, high-density lipoprotein-c immune FS, triglycerides FS, alkaline phosphatase, and low-density lipoprotein cholesterol Selectra were purchased from Response^®^-Diagnostic Systems, Holzheim, Germany, whereas rat feed ingredients and triton WR-1339 for hypercholesterolemia were purchased from a local market in Lahore. The superoxide dismutase (SOD) and glutathione peroxidase (GPx) required kits were purchased from Randox Laboratories Ltd., Crumlin, County Antrim, UK. Moreover, the fresh samples of oyster mushrooms were collected from farms of Kasur and Punjab, Pakistan, and white button mushrooms were collected from a local market in Lahore. After the collection of mushroom samples, they were placed in an ice box container and transported immediately to the food analysis laboratory of the Department of Food Science and Human Nutrition, at the University of Veterinary and Animal Sciences Lahore, for storage and subsequent analysis. Afterwards, the mushrooms were dried in a hot air dryer at 50 °C for 6 h followed by grinding to make fine powder. Afterwards, the mushroom powder was packed in polyethylene bags and stored for further use and analysis.

### 2.2. Preparation of Mushroom Extracts

For the preparation of the mushroom samples’ extracts, the weighed dried mushrooms were ground into fine powder mechanically followed by sieving prior to subjection to extraction. The powdered samples of both mushrooms (50 g) were extracted with 300 mL of 95% ethanol for 8 h using Soxhlet apparatus (Biocoction, Bengal, Kolkata) and residual solvent was removed by evaporation at 40 °C for 4 h using a vacuum in the rotatory evaporator (Heidolph Hei-VAP Core, Schwabach, Germany) by following the procedure as described by [14]. 

### 2.3. Animals’ Procurement, Housing, and Handling

For the animal model study using rats to explore therapeutic potential of mushrooms, 56 normal male adult rats weighing 150 ± 5 g and aged approximately 5–6 weeks were purchased from the Pakistan Council of Scientific and Industrial Research (PCSIR), Lahore, and given basal diet for 15 days in the animal shed for acclimatization. For rat trial, stainless-steel cages were developed in which animals were housed with wood litter bedding changed weekly to maintain hygiene along with disease prevention and also the cages’ temperature was maintained at 24 ± 2 °C. Additionally, fresh water and a standard diet were provided to the rats that were subjected to the dietary intervention trial. Before study trial initiation, the ethical approval (no. DR 374, dated 8 August 2022) for the execution of the trial and animal handling was obtained from the Institutional Ethical Committee, at the University of Veterinary and Animal Sciences, Lahore, Pakistan, that approved all required methods and procedures for conducting trial as well as analyzing the samples of the rats.

### 2.4. Experimental Design

**Figure 1:**
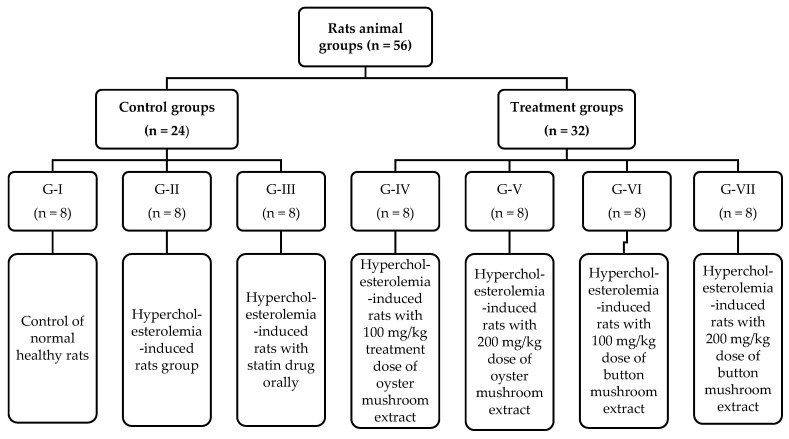
Diagram showing experimental design with animal groups subjected to different dietary mushroom intervention.

For the study to explain the effect of selected white button and oyster mushroom extracts on hypercholesterolemia and dyslipidemia, the rats were divided into 7 groups with each comprising 8 rats, i.e., G_1_ (negative control), G_2_ (positive control, HC rats with normal diet given), G_3_ (HC rats with statin drug orally given), and the next 4 treatment groups were G_4_ (HC rats with 100 mg/kg dose of oyster mushroom extract), G_5_ (HC rats with 200 mg/kg dose of oyster mushroom extract), G_6_ (HC rats with 100 mg/kg BW dose of white button mushroom extract), and G_7_ (HC rats with 200 mg/kg BW dose of white button mushroom extract), and detail of the rats plan is depicted in Figure 1.

### 2.5. Induction of Hypercholesterolemia in Rats

The hypercholesterolemia in rats was induced experimentally in 12 h fasting rats by providing high-fat diet and single intra-peritoneal injection of triton WR-1339 (300 mg/kg body weight dissolved in 0.89% saline solution) by following the procedure as described by [15]. After administration (48 h) of triton WR-1339, the rats exhibited elevated serum levels of total cholesterol and triglycerides. Moreover, the dietary supplementations of mushroom extracts with 100 and 200 mg dose were administered continuously from week 1 to week 4 after the induction of hypercholesterolemia; however, food intake and body weight of the rats were recorded on a weekly basis to check the impact of mushroom extract treatments on growth parameters such as feed intake and weight gain of the rats.

### 2.6. Collection of Blood Samples

After the completion of the research trial, the blood samples of rats provided mushroom extract were collected from the rat tail to estimate blood lipid profile, and assess antioxidant parameters of the rats. Before commencing blood sample collection, all necessary chemicals, surgical tools, and fluids required at the working site were provided and verified. The collection tubes for blood samples were labeled prior to the experiment, and the blood was collected in the appropriately labeled tube by following the guidelines of [16]. Accordingly, blood serum was separated by centrifuging at 1500× *g* for 15 min.

### 2.7. Serum Biochemical Analysis

At the termination of the dietary intervention of mushroom extracts to the rats, the collected blood samples were subjected to analysis to measure the biochemical parameters of total cholesterol (TC), low-density lipoprotein (LDL), high-density lipoprotein (HDL), and triglycerides (TG) with commercially available Abbott kits using Affinity I by following the guidelines of [8]. Blood glucose levels, liver function test, and renal function test parameters were estimated by the Randox kits method using Daytona Plus (Randox Laboratories Ltd., Crumlin, County Antrim, UK).

### 2.8. Estimation of Liver and Renal Function Markers

The activity of plasma transaminases, glutamate pyruvate transaminase (GPT), and glutamate oxaloacetate transaminase (GOT) were determined using the standard method by following the guidelines of [8]. The resultant oxoacids from transaminase reaction were assessed in an indirect manner by enzymatically converting them into their corresponding hydroxy acids. The alteration in NADH concentration that occurred concurrently was observed using spectrophotometric analysis at a wavelength of 340 nm. For the quantitative in vitro determination of transaminases, glutamate oxaloacetate transaminase (GOT) and glutamate pyruvate transaminase (GPT), in serum using the Randox kit method on the Daytona Plus apparatus, the buffer solution comprised 100 mmol/L tris buffer (pH 7.5) and 0.6 mol/L L-alanine solution. Afterwards, the fresh ddH_2_O was aspirated, and a new gain calibration was performed in flow cell mode on the apparatus. Then, ALT was selected on the run test screen, and a water blank was executed. Subsequently, 0.05 mL of the sample and 0.5 mL of the reagent were transferred into a test tube, thoroughly mixed, and aspirated into the analyzer at 37 °C. For the determination of plasma alkaline phosphatase (ALP) activity of rats with thymolphthalein monophosphate, the serum was mixed with 2-amino, 2-methyl, and 1-propanol (AMP) buffer at pH 8.5 with optimal temperature for analysis set at 37 °C. Accordingly, a 0.35 M 2-amino-2-methyl-1-propanol buffer was prepared by diluting the concentrated buffer 10-fold with water followed by preparation of fresh working standard by diluting 0.50 mL of the stock standard to 50 mL with 0.35 M 2-amino-2-methyl-1-propanol buffer (where 1 mL equaled 0.10 µmol of thymolphthalein). Afterwards, the 6 cuvettes were labelled, and 0, 0.20, 0.40, 0.60, 0.80, and 1.00 mL of the working standard were added to them, respectively. Then, 1 mL of buffered substrate was pipetted into appropriately labeled test tubes/cuvettes and placed in a water bath maintained at 37 ± 0.1 °C for 5 min. Afterwards, 0.10 mL of serum was added using a pipette before rinsing the pipette twice. Exactly 10 min after adding the serum, 5 mL of the color developer was added, followed by stoppering and mixing by inversion. The absorbance of the sample was taken at 590 nm against reagent blank, which was prepared by substituting water for serum. Afterwards, alkaline phosphatase activity was determined using the standard curve and sample absorbance. The levels of creatinine, markers of kidney function tests, were measured by the Randox kits (Randox Laboratories Ltd., Crumlin, UK) method (Daytona Plus) by following the protocol of [17]. After blood sampling of the rats for the determination of creatine, urea, and uric acid, the serum was separated and colorimetric method was used for the quantitative determination of creatinine in serum, 50 µL of dH_2_O was pipetted into a cuvette for the reagent blank, while 50 µL of standard was added into another separate cuvette. Simultaneously, 50 µL of sample was placed in another cuvette. Each cuvette received 500 µL of working reagent followed by thorough mixing, all cuvettes were inserted into the blood chemistry analyzer holder for the determination of parameters, and all the analysis was performed at 37 °C.

### 2.9. Estimation of Antioxidant Biomarkers

For the estimation of antioxidant biomarkers, the different enzymes like catalase (CAT), superoxide dismutase (SOD), and glutathione peroxidase (Gpx) that work together to protect cellular components against damage caused by free radicals, were determined. Antioxidant biomarkers like superoxide dismutase (SOD), malondialdehyde (MDA), and glutathione peroxidase (GPx) were determined using the standard kit method. The superoxide dismutase is a well-known antioxidant enzyme. It protects the cell against reactive oxygen species (ROS) by scavenging the activity of O_2_ and hydrogen peroxide (H_2_O_2_). The SOD activity was estimated by following the protocol as described by [18]. For the analysis, the reaction mixture consisting of 500 μL plasma homogenate, carbonate solution (50 mM), and 17 μL adrenaline was taken. Afterwards, the adrenaline was oxidized to adrenochrome, a colored product that was detected at 480 nm. The rate at which adrenaline was inhibited in the samples was monitored for 180 s, whereas enzymatic activity was expressed in U/mL of protein in the sample. Additionally, the concentration of malondialdehyde (MDA), an oxidative stress marker of the rats, provided different levels for oyster and white button mushrooms and was determined by following [19]. For the analysis, we collected serum of 200 μL plasma homogenate and sodium dodecyl sulfate (SDS) and 750 μL of acetic acid and thiobarbituric acid (TBA). Afterwards, the mixture was incubated at 95 °C for 1 h and 30 min. The absorbance was measured at 532 nm and MDA tissue level was expressed as μmol MDA/L. The glutathione peroxidase is an important oxidative stress biomarker and its activity in blood samples was estimated by following the method of [19]. To perform the test, an assay mixture containing 0.5 mL of sodium phosphate buffer, 0.1 mL of 10 mM sodium azide, 0.2 mL of 4 mM reduced glutathione, and 0.1 mL of 2.5 mM H_2_O_2_ was prepared first. To this mixture, 100 μL of serum homogenate sample was added and total volume was made up to 2 mL by using distilled water. Afterwards, the mixture was incubated at 37 °C for 3 min and the reaction was finally terminated by adding 0.5 mL of 10% trichloroacetic acid (TCA) followed by centrifugation (1000 rpm for 10 min) to determine the residual glutathione content. Afterwards, supernatant was collected and to this, 4 mL of disodium hydrogen phosphate (0.3 M) solution and 1 mL of dithio-bisnitrobenzoic acid (DTNB) were added followed by color development that was read against a reagent blank (containing only the phosphate solution and DTNB reagent) at 412 nm. The enzyme activity was expressed as units/mg protein.

### 2.10. Histopathology Analysis

At the end of the research trial, the livers of the rats subjected to dietary intervention of mushroom extracts along with control were subjected to hematoxylin-eosin staining method to create histopathological analyses by following the guidelines of [20]. Accordingly for the analysis, the examination specimens were taken from each rat’s liver and placed in 10% formal saline solution for 24 h. Afterwards, raised tissue portions were assembled on glass slides, deparaffinized, and stained with hematoxylin and eosin for regular analysis under light microscope.

### 2.11. Statistical Analysis

All the collected data were statistically evaluated via statistical package for social sciences (SPSS) version 20 with values expressed as means ± SD. One-way analysis of variance (ANOVA) was performed to assess significance among the study parameters, where the Duncan multiple range test (DMRt) was performed on the significantly different parameters to assess the differences between the treatments and study parameters considered as indicative of significance (*p* < 0.05) by following the guidelines of [21].

## 3. Results

The primary objective of this study was to assess the hypolipidemic potential of oyster and white button indigenous mushroom extracts grown in Pakistan using a rat animal model, focusing on their ability to lower lipid levels and manage hyperlipidemia with the specific goal of determining their effectiveness in combating oxidative stress and reducing the damage caused by free radicals.

### 3.1. Effect of Oyster and White Button Mushrooms on Plasma Lipid Profile

The results (Table 1) display different levels of various parameters (mg/dL) of the lipid profile of the rats’ blood in the different treatments groups of experimental rats that were provided with various levels of oyster and white button mushrooms, reporting significant variations due to provision of dietary mushroom treatments (*p* < 0.05). Means for total cholesterol indicated that lowest value for total cholesterol was reported in G_3_ (HC rats with statin drug orally) as 48.5 ± 1.4 followed by G_1_ (negative control) as 58.8 ± 1.5 and highest was reported in G_2_ (positive control group) as 72.7 ± 2. For the triglycerides level of rat blood results, the lowest triglycerides value was observed in G_1_ as 91.3 ± 1.6 followed by 78.8 ± 1.4 in G_3_ 107.5 ± 1.6 in G_5_, whereas the highest value was documented in G_2_ as 144.1 ± 1.9. Considering the results of the dietary treatments groups, the first treatment group was hypercholesterolemic rats that were provided with 100 mg extract of *Pleurotus ostreatus* whereas the second group of rats were given 200 mg dose extract of *Pleurotus ostreatus*. The group 3 consisted of hypercholesterolemic rats given 100 mg extract of *Agaricus bisporus* and the fourth group was hypercholesterolemic rats treated with 200 mg extract dose of *Agaricus bisporus*. Considering the mushroom treatments groups, the lowest total cholesterol was reported in G_5_ as (63.12 ± 2.52), followed by G_7_ as (64.11 ± 2.34), whereas the highest cholesterol content was documented in G_4_ as 72.06 ± 1.82. Similarly, for the triglycerides, the lowest values were reported in G_5_ and G_7_, which were lower than G_4_ and G_6_, which relates with the dose-dependent responses; however, the mushroom varieties were performing almost similarly regarding hypolipidemic behavior.

#### 3.1.1. Total Cholesterol of Rats

The hypercholesterolemic group exhibited significantly higher TC levels compared to the healthy rats group (72.74 ± 2.06 vs. 58.82 ± 1.53, *p* < 0.05), indicating elevated cholesterol levels in the hypercholesterolemic state. Both, the hypercholesterolemic rats with statin drug group and the hypercholesterolemic rats group treated with 200 mg dose extract of *Pleurotus ostreatus* showed significant reductions in total cholesterol levels (48.53 ± 1.43 and 63.12 ± 2.52, respectively) compared to the hypercholesterolemic control group. Similarly, the group of hypercholesterolemic rats treated with 100 mg dose extract of *Agaricus bisporus* and the group of hypercholesterolemic rats given 200 mg extract of *Agaricus bisporus* also demonstrated lower TC levels (66.32 ± 2.2 and 64.11 ± 2.3, respectively) as compared to the control group of hypercholesterolemia rats. 

#### 3.1.2. Triglycerides Level

Considering the results for triglycerides of rats subjected to different dietary treatments, the hypercholesterolemic rats group exhibited significantly higher TG levels compared to the control group (144.1 ± 1.9 vs. 91.3 ± 1.6, *p* < 0.05), indicating elevated triglyceride levels in the hypercholesterolemic induced state. However, both the drug control group and treatment group with 200 mg extract dose of *Pleurotus ostreatus* showed significant reductions in TG levels (78.8 ± 1.4 and 107.5 ± 1.6, respectively) compared to the hypercholesterolemic group. Similarly, the treatment group with *Agaricus bisporus* 100 mg group and treatment group with 200 mg *Agaricus bisporus* group also demonstrated lower TG levels (136.1 ± 1.5 and 128.3 ± 2.3, respectively). 

#### 3.1.3. High-Density and Low-Density Lipoproteins

The results regarding high-density and low-density lipoproteins, hypercholesterolemic rats group 2 showed significantly lower levels of HDL compared to normal rats group (21.2 ± 1.5 vs. 32.5 ± 1.5, *p* < 0.05), indicating the lipid profile in the hypercholesterolemic rats is varied. The statin drug was used in the control group and mushroom extracts were used in the treatment groups. The HC group showed significantly lower levels of HDL (21.2 ± 1.5) compared to the healthy group (32.52 ± 1.52, *p* < 0.05), indicating higher values for the parameters. However, the HC + statin and HC + PO groups demonstrated an increase in HDL levels (21.8 ± 1.78, 23.5 ± 1.42, 27.23 ± 1.69, and 21.09 ± 1.73) compared to HC, suggesting the beneficial effects of both statin therapy and mushrooms extract supplementation on HDL levels. In terms of LDL levels, the HC group showed significantly higher levels compared to the healthy rat group (23.32 ± 1.92 vs. 11.18 ± 2.04, *p* < 0.05). However, both HC + statin and HC + PO groups demonstrated a significant decrease in LDL levels (12.32 ± 1.92, 21.5 ± 1.83, 10.21 ± 1.23, and 22.42 ± 1.54) compared to HC, indicating the effectiveness of statin therapy and mushroom extract supplementation in lowering LDL levels. These findings are consistent with previous studies that have reported the lipid-modifying effects of statins and certain plant oils on HD levels in rat animal studies.

### 3.2. Effects of Mushrooms Extracts on Plasma Glucose Level, and Liver and Kidney Function

The Table 2 represents results of different parameters such as glucose, bilirubin, and creatinine levels of rats subjected to dietary intervention of *P. ostreatus* and *A. bisporus* for control, hypercholesterolemic (HC), and treatment groups. Means glucose level in G_7_ (HC rats provided 200 mg/kg BW dose of white button mushroom extract) indicated the non-significant 80.25 ± 5.62, followed by G_5_ (HC rats given 200 mg/kg body weight (BW) dose of oyster mushroom extract) as 85.22 ± 6.61 whereas elevated levels of glucose was reported in G_2_ (positive control) as 118.21 ± 10.74. 

The groups G_4_ and G_6_, treated with 100 mg/kg dose of oyster mushroom and button mushroom, indicated that both extract dosage are non-significant to group 2. Oyster and white button mushroom extracts at 200 mg/kg dose in G_5_ and G_7_, respectively, exhibit significantly lower glucose levels (85.22 ± 6.61, 80.25 ± 5.62) compared to the hypercholesterolemia-induced control group G_2_ (118.21 ± 10.74). Results show that white button mushrooms have stronger antidiabetic potential than oyster. Results also show that both mushrooms showed significant impact on blood glucose management. There are no significant differences reported in bilirubin levels among all treatment groups. Serum creatinine levels in group G_2_ (0.63 ± 0.13) has significance with group 5 (0.41 ± 0.11). Overall, the results suggest that the administration of oyster mushroom and button mushroom extracts at higher doses (200 mg/kg) have beneficial effect on serum bilirubin, and creatinine and glucose levels in hypercholesterolemic rats.

Results in (Table 3) display the effects of oyster and white button mushrooms on liver and kidney function tests in hypercholesterolemic rats which indicated that dietary provision of oyster and white button mushroom extracts reduced ALP levels compared to the hypercholesterolemia-induced control (G_2_), but the change was nonsignificant (*p* < 0.05). Additionally, no notable differences in GOT and GPT levels were observed across all groups, suggesting minimal impact of the dietary intervention on the rats. Overall, the hypercholesterolemia has a mild impact on the rat liver enzymes, with ALP showing significant increase by the dietary intervention. However, the supplementation with oyster and white button mushroom extracts positively influence ALP levels, suggesting potential liver protection by the extracts’ supplementation.

### 3.3. Enzymatic Markers of Antioxidant Activity

**Figure 2:**
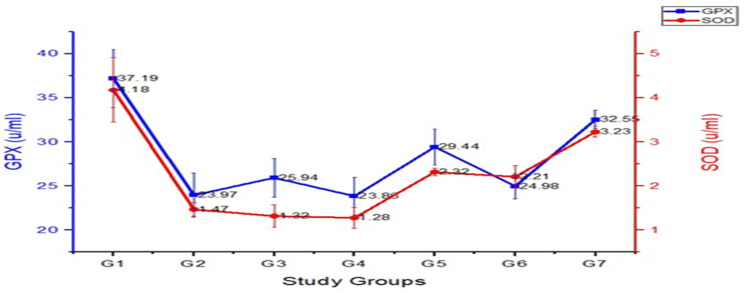
The graph shows antioxidant parameters, i.e., GPx = glutathione peroxidase and superoxide dismutase, relating to G_1_ (negative control), G_2_ (positive control), G_3_ (hypercholesterolemia-induced rats with statin drug orally), G_4_ and G_5_ (hypercholesterolemia-induced rats provided @ 100 and 200 mg/kg BW dose of oyster mushroom extracts), and G_6_ and G_7_ (hypercholesterolemia-induced rats given @ 100 and 200 mg/kg BW dose of white button mushroom extracts).

Figure 2 represents the results of the present study, indicating significant differences in the levels of antioxidant enzymes (SOD and GPx) among the different experimental groups of rats provided with oyster and white button mushroom extracts. Means for SOD values indicated that among treatments, the lowest SOD value observed was 1.28 ± 0.24 in G_4_ (HC rats provided @ 100 mg/kg body weight (BW) dose of oyster mushroom extract), followed by 1.32 ± 0.26 in G_3_ (HC rats provided with statin drug orally), whereas the highest value was documented in G_0_ (negative control) as 4.18 ± 0.73.

Superoxide dismutase (SOD) is an important antioxidant enzyme involved in the defense against reactive oxygen species in living organisms. In the hypercholesterolemic (HC) groups, the SOD levels were significantly lower compared to the control group, suggesting reduction in the antioxidant defense system of rats. However, in the treatment group as HC + PO (100 mg dose) and HC + AB (100 mg dose), the SOD levels were significantly higher than the HC group, indicating that the administration of oyster and white button mushroom extracts increased the activity of SOD, potentially enhancing the antioxidant capacity of the treated rats. Glutathione peroxidase (GPx) is another antioxidant enzyme that helps protect cells from oxidative damage. The present study findings for hypercholesterolemic (HC) group showed significantly lower GPx levels compared to the healthy control rats, indicating impaired antioxidant function due to cholesterol induction in the rats. However, in treatment group with *Pleurotus ostreatus* dose and *Agaricus bisporus*, GPx levels were higher than the HC group, suggesting that the supplementation with oyster and white button mushroom extracts partially restored the activity of GPx, potentially improving the antioxidant capacity. Means for GPx values indicated that among the treatments, the lowest GPx value was observed as 23.86 ± 2.11 in G_5_, followed by 23.97 ± 2.52 in G_2_, and 24.98 ± 1.42 in G_6_, whereas the highest GPx value was documented in G_0_ as 37.19 ± 3.29. Overall, the results suggested that in hypercholesterolemic condition, induced oxidative stress is evidenced by decreased SOD and GPx levels. However, the supplementation of oyster and white button mushroom extracts appears to have beneficial effects by increasing the activity of antioxidant enzymes.

### 3.4. Lipid Peroxidation Markers

**Figure 3:**
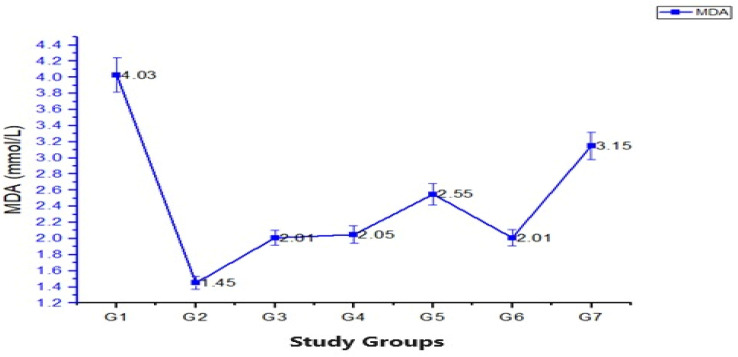
The graph shows the malondialdehyde levels of rats subjected to dietary mushroom extracts relating to G_1_ (negative control), G_2_ (positive control), G_3_ (hypercholesterolemia-induced rats with statin drug orally), G_4_ and G_5_ (hypercholesterolemia-induced rats provided @ 100 and 200 mg/kg BW dose of oyster mushroom extracts), and G_6_ and G_7_ (hypercholesterolemia-induced rats given @ 100 and 200 mg/kg BW dose of white button mushroom extracts).

Malondialdehyde (MDA) is a byproduct of lipid peroxidation and serves as a marker of oxidative stress. The hypercholesterolemic (HC) group exhibited significantly higher MDA levels compared to the healthy rats, indicating increased lipid peroxidation and oxidative damage. In contrast, the hypercholesterolemic rats treated with oyster and white button mushrooms showed lower MDA levels, suggesting that the administration of oyster and white button mushroom extract reduced lipid peroxidation and protected against oxidative stress. Means for MDA values for different treatments indicated highest value in G_2_ as 6.12 ± 0.89, showing that G_2_ rats had higher lipid peroxidation rate followed by G_7_ as 2.87 ± 0.05, then, G_4_ and G_5_, as 2.67 ± 0.31 and 2.55 ± 0.42, whereas the highest value was reported in G_3_ as 2.01 ± 0.03 (Figure 3). Overall, lipid peroxidation marker MDA showed that mushroom dose had reducing impact on MDA levels by reducing lipid peroxidation and protecting cellular damage.

### 3.5. Histopathology Analysis of Liver

**Figure 4:**
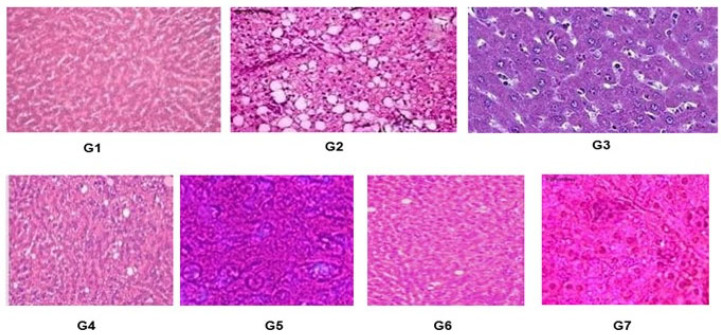
Histopathology of the liver tissues of rats of both control and treatment groups for 4 weeks. (G_1_) Control of normal healthy rats, (G_2_) control of hypercholesterolemia-induced rats, (G_3_) hypercholesterolemia-induced rats with statin drug orally, (G_4_) hypercholesterolemia-induced rats with 100 mg/kg treatment dose of oyster mushroom extract, (G_5_) hypercholesterolemia-induced rats with 200 mg/kg dose of oyster mushroom extract, (G_6_) hypercholesterolemia-induced rats with 100 mg/kg dose of button mushroom extract, and (G_7_) hypercholesterolemia-induced rats with 200 mg/kg dose of button mushroom extract.

The results in (Figure 4) showed histological changes of rats’ livers of the control and dietary treatment groups which indicate healthy control group (G_1_) exhibited standard liver histology, featuring hepatocytes arranged in a radial pattern around the central vein. The hepatocytes displayed characteristic size and morphology. G_2_ (hypercholesterolemic rats) had defective liver lobes and ambiguous hepatocyte structural boundaries whereas the livers of G_3_ (treated with statin drug) indicated histopathology findings as mild dysfunction. The treatment groups G_4_ and G_5_ treated with 100 mg/kg and 200 mg/kg oyster mushroom extract reported liver histology was inclined towards normal in the dose-dependent manner that reflected the potential of mushrooms as therapeutic agents. Similarly, treatment groups G_6_ and G_7_ treated with 100 and 200 mg/kg white button mushroom extract had a similar pattern for histology from hypercholesterolemia by increasing extract dose in the dose-dependent manner.

## 4. Discussion

The present study was conducted to explicate the hypolipidemic and antioxidant impact of two edible mushrooms in hypercholesterolemic rats. For this, rats were divided into different groups, which were healthy control, hypercholesterolemic, statin drug, white button extracts (100 and 200), and oyster mushroom extract (100 and 200) groups. Results indicated significant differences were observed in different measured parameters. Hypercholesterolemic rats had elevated total cholesterol (TC) levels compared to the healthy control group, whereas the statin group showed a significant decrease in TC levels, demonstrating the effectiveness of statins in lowering cholesterol. Similarly, white button mushroom extract and oyster mushroom extract groups exhibited lower TC levels, indicating their therapeutic potential in cholesterol-lowering in rats.

These results demonstrated a significant elevation in triglyceride (TG) levels in the hypercholesterolemic group compared to the healthy rats group, confirming the development of dyslipidemia in our experimental model. Administration of the statin to the hypercholesterolemic rat groups resulted in substantial reduction in TG levels, corroborating the established lipid-lowering effects of statin. Furthermore, our findings revealed that supplementation with white button extract and oyster extract in the hypercholesterolemic group led to a significant decrease in TG levels compared to the hypercholesterolemic group. This indicates the potential of these mushroom extracts to modulate TG metabolism and support the management of dyslipidemia. The observed lipid-lowering effects of white button and oyster mushroom extracts are in agreement with [8], who highlighted the bioactive components present in mushrooms contribute to their beneficial effects in lowering lipid profiles. These findings provide additional evidence for lipid-lowering properties of these mushroom extracts and support their potential application as natural interventions for management of dyslipidemia. Our study findings are also in agreement with [8] by demonstrating the significant reduction in TG levels in response to statin treatment and potential of white button and oyster mushroom extracts to modulate TG, thereby offering promising strategies for managing dyslipidemia.

Our study also reported that hypercholesterolemia is associated with lower levels of high-density lipoprotein (HDL), which is consistent with previous research findings. Interestingly, while the statin drug did not significantly improve HDL levels, both white button and oyster mushroom extract groups showed higher HDL levels. These findings suggest that incorporating these mushroom extracts in the diet may have a beneficial effect in increasing HDL cholesterol. These results are in line with findings of [22] who highlighted lipid-modulating properties of these mushroom extracts. Overall, our study supports the potential of these extracts in improving HDL cholesterol and warrants further investigation in dyslipidemia management. The present study also observed higher levels of LDL and vLDL cholesterol in the hypercholesterolemic group, which is in agreement with previous research highlighting the association between hypercholesterolemia and elevated LDL and vLDL levels. The administration of statins demonstrated significant efficacy in reducing LDL and vLDL cholesterol, validating their well-established role in lipid management. Additionally, the supplementation of white button and oyster mushroom extracts exhibited promising effects in lowering LDL and vLDL cholesterol, indicating their potential as natural interventions for dyslipidemia. These findings are consistent with [23] emphasizing the cholesterol-modulating properties of statins and selected mushroom extract. Overall, the findings of this study supported by previous researches [24] by demonstrating the detrimental effects of hypercholesterolemia on lipid profile parameters and potential beneficial effects of statin drugs and extracts of oyster and white button mushrooms in improving lipid profiles. The present study findings for lipid profile results are also supported by [25,26].

The analysis of liver function enzymes, including glutamic oxaloacetic transaminase (GOT), glutamic pyruvic transaminase (GPT), and alkaline phosphatase (ALP), provides insights into liver function and potential liver damage. The present study findings indicated that levels of GOT and GPT were found to be elevated in the hypercholesterolemic-induced group compared to the control group, indicating liver injury and dysfunction. However, treatments with mushroom extracts resulted in reduced levels of GOT and GPT, suggesting a potential protective effect of mushroom extracts. Additionally, the levels of ALP, another liver enzyme, were elevated in the hypercholesterolemia-induced group compared to the control group, indicating impaired liver function; however, the treatments with mushroom extracts showed a decreasing trend towards reducing the levels of ALP, suggesting a potential improvement in liver function. These findings align with [27,28] who reported the hepatoprotective properties of mushrooms and their ability to mitigate liver damage. The bioactive compounds present in mushroom extracts like polysaccharides and phenolics have been shown to exhibit hepatoprotective effects by reducing inflammation and oxidative stress in the liver. These findings are consistent with previous research that has linked dyslipidemia and hypercholesterolemia to liver damage and increased levels of liver enzymes. Similarly, one of the researchers group [29] reported the beneficial effects of mushroom consumption on liver health and the reduction of ALP levels. The observed trends in the liver enzyme levels in response to mushroom extract treatments support the notion that mushrooms may have a positive impact on liver function and help alleviate liver damage caused by dyslipidemia. These results are consistent with [30] and support the use of mushroom extracts as a natural intervention for improving liver health in conditions associated with dyslipidemia. Our findings are also supported by the study in [31], which also showed significant impact of edible mushrooms on liver and kidney functions.

The results obtained from the analysis of antioxidant parameters in different treatment groups provide valuable insights into the potential effects of white button and oyster mushroom extracts on oxidative stress and antioxidant defense mechanism in rats. In this study, the levels of superoxide dismutase (SOD) activity were found to be significantly lower in the hypercholesterolemic group compared to the control, indicating a compromised antioxidant defense system in hypercholesterolemic rats. Upon treatment with oyster and white button mushroom extract, the SOD activity was increased, suggesting these mushroom extracts have the ability to enhance the antioxidant capacity of rats. These findings are in agreement with [16,32] who reported the antioxidant properties of mushrooms and their ability to enhance the activity of antioxidant enzymes. Furthermore, the levels of glutathione peroxidase (GPx) were significantly lower in the hypercholesterolemia-induced group compared to the control, indicating a compromised antioxidant defense system and increased oxidative stress. However, treatment with oyster mushroom extract and button mushroom extract led to an increase in GPx levels. These findings are supported by [33,34] who have reported the antioxidant effects of mushroom extracts and their ability to improve GPx activity. Regarding malondialdehyde (MDA) levels, an indicator of lipid peroxidation, the hypercholesterolemia-induced group exhibited significantly higher MDA levels compared to the control group, indicating increased oxidative damage to lipids; however, treatments with oyster mushroom and white button mushroom extracts resulted in decreased MDA levels, suggesting the potential of these mushroom extracts to mitigate lipid peroxidation and reduce oxidative stress. These findings are consistent with [35,36] who have reported the antioxidant properties of these mushroom extracts. The presence of bioactive compounds such as phenolic, flavonoids, and polysaccharides in mushrooms contribute to their antioxidant effects, The observed increase in antioxidant enzyme activity and decrease in lipid peroxidation. Bioactive compounds, especially phenolic compounds, protect cellular components, such as DNA, lipids, and proteins, from oxidative damage. Overall, the present study provides evidence supporting the antioxidant effects of mushroom extracts, specifically, oyster mushroom and button mushroom extracts, in mitigating oxidative stress and enhancing antioxidant defense. These findings are further supported by existing literature of [37,38] on the beneficial effects of mushroom consumption on oxidative balance and reinforce the potential use of mushroom extracts as natural antioxidants in managing conditions associated with oxidative stress.

Histopathological analysis of hepatic tissue demonstrated that administration of extract of oyster and white button mushrooms provided protection to hepatocytes against the induced liver damage induced by triton WR 1339 injection. Both mushroom varieties contain phenolics, triterpenes, polysaccharides, and peptides which are the main classes of compounds which could be responsible for the hepatoprotective activity of the mushroom extracts [39,40]. These findings are supported by the studies that showed that being rich in polysaccharides and phenolics, oyster mushrooms show hepatoprotective properties [41]. Similar results were obtained in another study in which *P. ostreatus* mycelium was tested against injury induced by thioacetamide in mice [42]. Following previous studies, the present study results showed that both mushroom extracts have hepatoprotective qualities against liver injury induced by chemical substances and could be used as a healthy dietary intervention.

## 5. Conclusions

The present study demonstrates the potential of indigenous mushroom extracts in reducing dyslipidemia in hypercholesterolemic rats. The extracts significantly lowered plasma cholesterol and triglyceride levels after a 28-day administration, whereas the treatments (@ 200 mg/kg BW) group showed greater efficacy in improving the lipid profile. Additionally, the mushroom extracts exhibited hypoglycemic and antioxidant properties; however, enzyme profiles of the rats were not significantly altered indicating that the effects of the extracts were not mediated through enzyme activity changes. The presence of bioactive poly phenolic compounds and polysaccharides indicated both mushroom hepatoprotective potential as a therapeutic agent. Overall, the study reports therapeutic potential of the oyster and white button mushroom extracts for managing dyslipidemia using a rat animal model study.

## Figures and Tables

**Table 1:** Effects of oyster and white button mushrooms on plasma lipid profiles of hypercholesterolemic rats.

Parameters (mg/dL)	G_1_	G_2_	G_3_	G_4_	G_5_	G_6_	G_7_
Total cholesterol	58.82 ± 1.53 ^c^	72.74 ± 2.06 ^a^	48.53 ± 1.43 ^d^	72.06 ± 1.82 ^a^	63.12 ± 2.52 ^b^	66.32 ± 2.22 ^b^	64.11 ± 2.34 ^b^
Triglycerides	91.32 ± 1.62 ^f^	144.1 ± 1.91 ^a^	78.83 ± 1.46 ^e^	138.6 ± 2.09 ^b^	107.5 ± 1.62 ^d^	136.1 ± 1.52 ^b^	128.31 ± 2.36 ^c^
High-density lipoprotein	32.52 ± 1.52 ^a^	21.2 ± 1.56 ^c^	21.8 ± 1.78 ^c^	23.5 ± 1.42 ^c^	27.23 ± 1.69 ^b^	21.09 ± 1.73 ^c^	21.82 ± 1.71 ^c^
Low-density lipoprotein	11.18 ± 2.04 ^c^	23.32 ± 1.92 ^a^	12.32 ±1.92 ^b,c^	21.5 ± 1.83 ^a^	10.21 ± 1.23 ^c^	22.42 ± 1.54 ^a^	15.04 ± 1.22 ^b^
Very-low-density lipoprotein	20.9 ± 1.54 ^c^	30.9 ± 2.12 ^a^	17.1 ± 1.34 ^d^	29.5 ± 1.61 ^a^	20.7 ± 2.24 ^c^	25.24 ± 1.52 ^b^	24.21 ± 1.35 ^b^

All data expressed as means ± SD. Means superscripted with different letters are significantly different (*p* < 0.05) whereas G_1_ (negative control), G_2_ (positive control), G_3_ (hypercholesterolemia-induced rats with statin drug orally), G_4_ and G_5_ (hypercholesterolemia-induced rats given @ 100 and 200 mg/kg BW dose of oyster mushroom extract), and G_6_ and G_7_ (hypercholesterolemia-induced rats given @ 100 and 200 mg/kg BW dose of white button mushroom extracts).

**Table 2:** Effects of extract of oyster and white button mushrooms on plasma glucose level, bilirubin and creatinine levels (mg/dL).

Parameters	G_1_	G_2_	G_3_	G_4_	G_5_	G_6_	G_7_
Glucose	106.04 ± 4.71 ^a^	118.21± 10.74 ^a^	100.13 ± 5.23	115.34 ± 8.42	85.22 ± 6.61 ^b^	114.83 ± 7.52	80.25 ± 5.62 ^b^
Bilirubin	0.28 ± 0.10	0.32 ± 0.11	0.25 ± 0.14	0.29 ± 0.12	0.26 ± 0.12	0.29 ± 0.11	0.31 ± 0.14
Creatinine	0.54 ± 0.12 ^a^	0.63 ± 0.13 ^a^	0.45 ± 0.21 ^b^	0.55 ± 0.11 ^a^	0.41 ± 0.11 ^b^	0.56 ± 0.21 ^a^	0.54 ± 0.21 ^a^

All data expressed as means ± SD. Means superscripted with different letters are significantly different (*p* < 0.05) whereas G_1_ (negative control), G_2_ (positive control), G_3_ (hypercholesterolemia-induced rats with statin drug orally), G_4_ and G_5_ (hypercholesterolemia-induced rats given @ 100 and 200 mg/kg BW dose of oyster mushroom extract), and G_6_ and G_7_ (hypercholesterolemia-induced rats given @ 100 and 200 mg/kg BW dose of white button mushroom extracts).

**Table 3:** Effects of oyster and white button mushroom extracts on plasma enzyme profile.

Parameters (U/L)	G_1_	G_2_	G_3_	G_4_	G_5_	G_6_	G_7_
Glutamic oxaloacetic transaminase (GOT)	37.0 ± 3.72 ^b^	40.8 ± 7.64 ^a^	39.10 ± 4.52 ^a^	39.8 ± 7.62 ^a^	39.1 ± 5.92 ^a^	38.8 ± 5.32 ^b^	38.7 ± 5.22 ^b^
Glutamic pyruvic transaminase (GPT)	35.2 ± 6.12	42.1 ± 5.71	39.12 ± 5.45	40.1 ± 4.64	35.6 ± 5.81	41.7 ± 5.12	37.8 ± 7.22
Alkaline phosphatase (ALP)	122.4 ± 7.71 ^a,b^	132.4 ± 6.12	126.41 ± 4.11	131.6 ± 7.82	111 ± 7.51 ^b^	130.7 ± 5.22	115.8 ± 13.62 ^a^

All data expressed as means ± SD. Means superscripted with different letters are significantly different (*p* < 0.05) whereas G_1_ (negative control), G_2_ (positive control), G_3_ (hypercholesterolemia-induced rats with statin drug orally), G_4_ and G_5_ (hypercholesterolemia-induced rats given 100 and 200 mg/kg BW dose of oyster mushroom extract), and G_6_ and G_7_ (hypercholesterolemia-induced rats given 100 and 200 mg/kg BW dose of white button mushroom extracts).

## Data Availability

The original contributions presented in the study are included in the article, further inquiries can be directed to the corresponding author.
